# Evidence for diurnal periodicity of earthquakes from midnight to daybreak

**DOI:** 10.1093/nsr/nwy117

**Published:** 2018-10-08

**Authors:** Jinlai Hao, Jinhai Zhang, Zhenxing Yao

**Affiliations:** Key Laboratory of Earth and Planetary Physics, Institute of Geology and Geophysics, Chinese Academy of Sciences, Beijing 100029, China

**Keywords:** periodicity of earthquakes, time-frequency analyses, ocean tide, earth tide, Local Mean Time

## Abstract

Previously, inadequate earthquake catalogs and the lack of method made it challenging, if not impossible, to determine the dominant period of earthquake occurrence. With the advent of densely distributed seismic stations in Japan since 2002, 410 642 M1.0+ earthquakes have been cataloged under the mainland within 30-km depth, which provide a superb dataset to identify the periodicity of earthquakes. We processed this catalog using time-frequency analyses and daily stacking, which are powerful in extracting weak periodical signal from strong random noises. We concentrated on the time sector from 0:00 to 6:00 (i.e. from midnight to daybreak), which is a reliable time window for much higher detectability of weak earthquakes, since it has the lowest affects from cultural noises. We successfully observed two apparent periodicities of 12- and 24-hour, which are much smaller than the fortnightly periodicity presented previously in the literature. Synthetic earth tides, after intentionally ignoring the contribution from the Moon, present similar dominant periods as the earthquakes. This may indicate that the dominant period of earthquakes is statistically associated with the Sun rather than the Moon. The daily stacking number of earthquakes using a 15-minute or 1-hour interval shows a peak around 1:30, rather than usually expected 3:00 to 4:00. In addition, bigger earthquakes show more evident variations in the stacking results, and the trend is very consistent for various lower limits of earthquake magnitude from M1.0 to M4.0. These discoveries settled the disputes on the existence of the periodicity of earthquakes since 1886 and may open a window to unravel the mystery of earthquakes.

## INTRODUCTION

The motions of the Sun and Moon cause tidal deformations of the Earth, and the resulting changes of the stress state in the lithosphere may trigger earthquakes. Numerous studies have investigated this phenomenon, termed tidally triggered earthquakes going as far back as 1886 [1–5], resulting in an intense debate on this subject, often focusing on problems introduced from inadequate earthquake catalogs [6–9]. Now, with the increasing number of seismic networks and improved earthquake catalogs, recent studies [10–19] have been able to statistically identify correlations between the rate of the phenomena called tremor and tidal forcing. Fortnightly modulation of tremor and low-frequency earthquakes was recently identified using several millions of earthquakes near the San Andreas Fault [20]. It was also found that very large earthquakes tend to occur near the time of the maximum tidal stress amplitude, but this tendency was not obvious for small earthquakes [21]. However, the earthquake period is still a traditional and disputable topic in seismology. Apparent weekly and daily earthquake periodicities are detected in the Western USA [22], which may suggest that the ambient noise from human activities, including the ground traffic, operating machineries and so on, is probably the major cause of the periodicities. The ambient noise from human activities strongly affected the detection of the earthquake periodicity, especially using the catalog of small-magnitude earthquakes.

**Figure 1:**
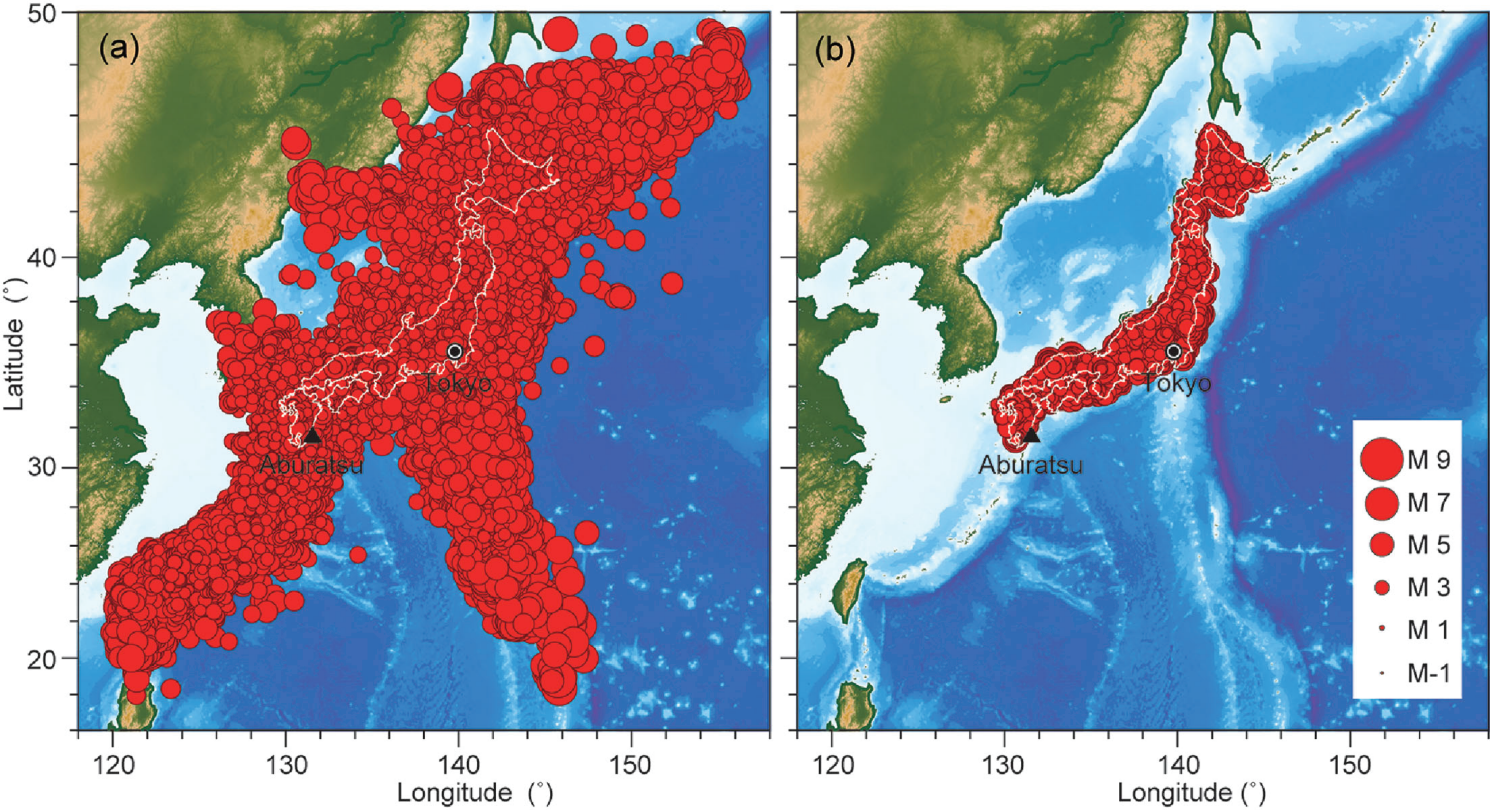
The spatial distribution of earthquakes in Japan and its adjacent areas. (a) Complete catalog from 3 June 2002 to 4 July 2018. (b) Selected catalog that has a magnitude of M1.0+ under the mainland according to Nanjo *et al.* (2010). The maximum depth of the selected earthquake is 30 km. The total number of events is 2 560  811 in (a) and 410 642 in (b), respectively. The symbol size represents earthquake magnitude.

According to the Gutenberg-Richter frequency-magnitude law [23,24], the number of small earthquakes is much bigger than that of large earthquakes. This indicates that we could extract the statistical periodicity if the number of small earthquakes is large enough after long-term continuous observation. The local catalog of earthquakes in Japan has been continuously recorded by high-precision seismometers within densely distributed networks since 3 June 2002 (http://www.hinet.bosai.go.jp/topics/JUICE/?LANG=en). The Japan Meteorological Agency (JMA) determines earthquakes’ hypocenter locations and their magnitudes. We applied the JMA unified earthquake catalog in this study [25]. It accumulated 2 560 811 events in total until 4 July 2018 (Fig. 1a). This is the best catalog for us to determine the period of earthquakes, if the period of earthquakes exists and is detectable. The JMA unified earthquake catalog included lots of events that are far from the Japanese mainland. Because the epicenters of these earthquakes are too far away from the dense seismic arrays, some smaller earthquakes could not be successfully identified, especially when the background noise is strong around the seismic stations (Fig. 1a). Nanjo *et al.* (2010) suggested using M1.0+ for the Japanese mainland to guarantee the completeness of magnitudes [26]. Here, we selected the earthquakes under the Japanese mainland with the depth less than 30 km and the selected catalog contains 410 642 M1.0+ earthquakes (Fig. 1b), without refining [27] or declustering [28] on the aftershocks.

The lack of data-processing methods for analysing the earthquake catalog is another reason for the debate on the existence of earthquake periodicity. We apply time-frequency analyses [29] and stacking to extract the dominant period from the earthquake catalog. Our numerical experiments on a synthetic earthquake catalog show that these tools can extract a weak periodical signal from extremely strong random noises (Supplementary Fig. 1a–d, available as Supplementary Data at *NSR* online) and a visible period emerges exactly at the given period in the results of time-frequency analyses (Supplementary Fig. 1e, available as Supplementary Data at *NSR* online). After stacking the results, we can further identify the weak periodical signal with a higher signal-to-noise ratio at a higher temporal resolution (Supplementary Fig. 1f, available as Supplementary Data at *NSR* online). Unfortunately, the parameter selection of the time interval for counting the number of earthquakes is essential for successful extraction of the weak periodical signal. A time interval that is too small would introduce many zero values in some time intervals, especially during a seismic quiet spell; in contrast, a time interval that is too big may not pick up the weak periodical signal from the currently available catalog due to the limited total number of earthquakes. After many tests, we selected 1 hour as the time interval, which could reduce the total number of zero values that appeared in some time intervals and can successfully extract the dominant periods.

According to universal gravitation, the tidal force on the Earth caused by the Moon is about 2.2 times larger than the Sun's tidal force. If one assumes, therefore, that the largest amplitude tidal forces are the primary triggering agent for earthquakes, tidally triggered earthquakes are more likely to be caused by the Moon. However, it has yet to be confirmed whether the tidal force amplitude is the primary triggering agent. Previous studies mainly concentrate on the relationships between earthquakes and tides (the earth tide and/or ocean tide) but do not separately consider the contributions from the Sun and the Moon. In this paper, we investigate the contributions from the Sun and the Moon individually. Our work shows that the earthquakes have two dominant periods of 12 and 24 hours; interestingly, both are associated with the Sun rather than the Moon.

**Figure 2:**
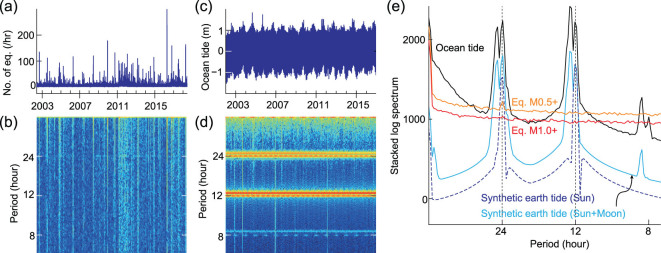
Comparison of dominant periods of earthquakes, ocean tide and synthetic earth tides. (a) Number of M0.5+ earthquakes within hourly bins from June 2002 to July 2018; (b) time-frequency spectrum of (a); (c) the variation of ocean-tide heights at Abratru from June 2002 to January 2017; (d) the time-frequency spectrum of (c); (e) comparison of the stacked spectrum.

## RESULTS

### Diurnal period from midnight to daybreak

Our time-frequency analyses of the M0.5+ earthquakes under the Japanese mainland (Fig. 1b) show a clear 24-hour dominant period (Fig. 2b and e), which correlates with the diurnal period. We also observe an additional period at 12 hours, which correlates with the Sun's semi-diurnal period, although its amplitude is much lower than that of the 24-hour period. Similarly, the M1.0+ earthquakes also exhibit a 24-hour period and a 12-hour period (Fig. 2e). According to Atef *et al.* [22], weekly and daily activities, such as the subway, ground traffic and industrial productions, may apparently reduce the detectability of weak earthquakes. Therefore, we concentrated on the time sector from 0:00 to 6:00 (i.e. from midnight to daybreak), which is a reliable time window for much higher detectability of weak earthquakes, since it has the lowest affects from cultural noises. We further collected the catalog only within this time sector to avoid the strong cultural noises from 6:00 to 24:00. If we can detect an apparent period of 6 hours using the collected catalog only within 0:00 to 6:00, we may infer the existence of a diurnal period of the earthquakes.

**Figure 3:**
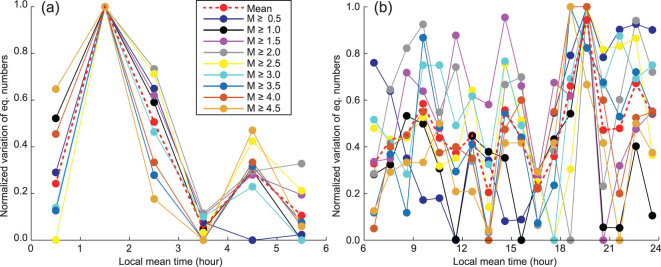
Normalized curve of daily stacked number of earthquakes using a 1-hour time interval. (a) Time sector from 0:00 to 6:00; (b) time sector from 6:00 to 24:00. The original curves of the daily stacking number of earthquakes are shown in Supplementary Fig. 2, available as Supplementary Data at *NSR* online.

**Figure 4:**
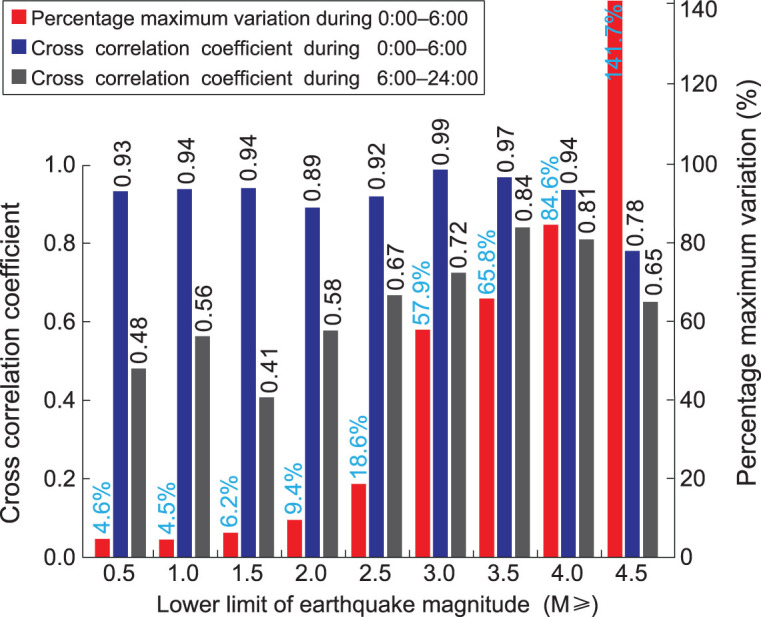
The cross-correlation coefficient and percentage maximum variation for each given lower limit of the earthquake magnitude. The cross-correlation is between the normalized curve of the daily stacking number of earthquakes and the averaged curve of all normalized curves within the given time sector. The percentage maximum variation is calculated by $(Max - Min)/Min$ of the daily stacking number of earthquakes within the given time sector.

As shown in Fig. 3 and Supplementary Fig. 2, available as Supplementary Data at *NSR* online, the daily stacking number of earthquakes within each hour in the time sector from 0:00 to 6:00 exhibits strong coherency for various lower limits of earthquake magnitude; in contrast, that from 6:00 to 24:00 exhibits almost no coherency. As shown in Fig. 4, the cross-correlation coefficient between each normalized curve shown in Fig. 3 and the averaged curve is generally bigger than 0.92 for M0.5+ to M4.0+ (except 0.89 for M2.0+) within the time sector from 0:00 to 6:00, and half of them are not smaller than 0.94; in contrast, the cross-correlation coefficient is generally smaller than 0.78 for M0.5+ to M4.0+ (except 0.84 for M3.5+) within the time sector from 6:00 to 24:00, and half of them are not bigger than 0.58. This indicates that the earthquakes exhibit stronger periodicity in the time sector from 0:00 to 6:00, compared with the time sector from 6:00 to 24:00. The percentage variation in the stacking number shows a rapidly rising trend with increasing lower limits of earthquake magnitude, as shown in Fig. 4. For M0.5+ to M2.0+, its value is not bigger than 10%; for M2.5+ to M4.5+, its value boosts from 19% to 142%. This significant trend indicates that bigger earthquakes have more evident periodicity. Therefore, we can conclude that the extracted dominant period is reliable and reflects the intrinsic characteristics of earthquakes.

### Unique dominant period

**Figure 5:**
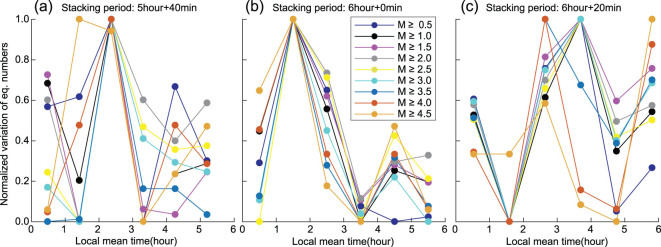
Normalized curve of stacking number of earthquakes for the time sector from 0:00 to 6:00. The time period used for stacking is 5 hours + 40 minutes (a), 6 hours (b) and 6 hours + 20 minutes (c). Only the time sector from 0:00 to 6:00 is considered in each day for this experiment, since the catalog of small-magnitude earthquakes has a high reliability without the affect of strong ambient noises due to human activities from 6:00 to 24:00.

**Figure 6:**
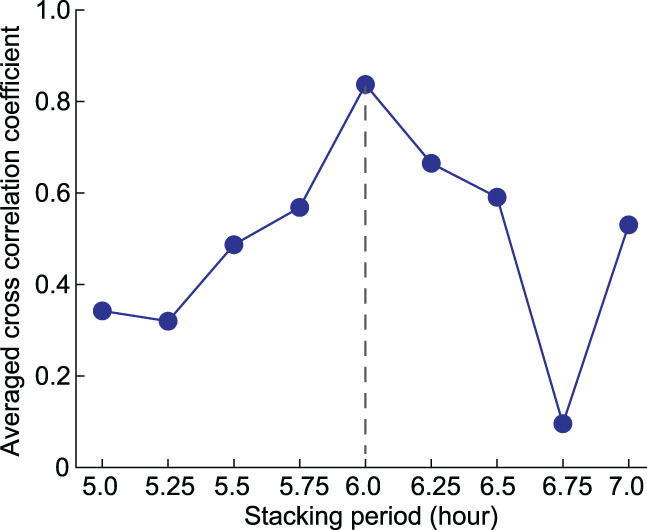
The averaged cross-correlation coefficient of the normalized curve of the daily stacking number of earthquakes. A given time period for stacking (e.g. 5 hours + 15 minutes) is first given to generate normalized curves (similar to Figs 3a and 5b); then, the averaged cross-correlation coefficients of these curves would be shown as a blue dot for the given time period; then, using a new time period for stacking (e.g. 5 hours + 30 minutes) and repeating the above process would generate a new blue dot. The given time periods range from 5 to 7 hours with an interval of 15 minutes.

**Figure 7:**
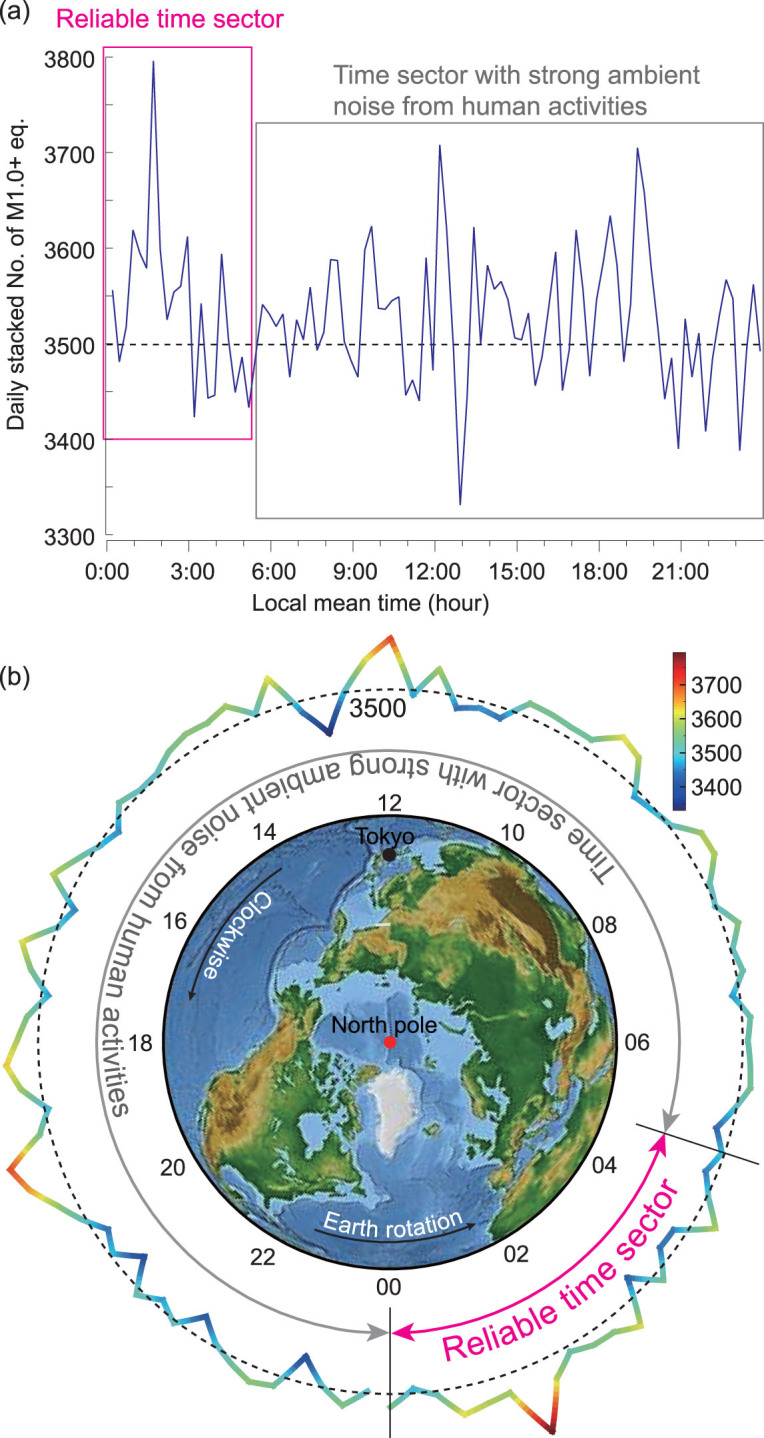
Daily stacking number of M1.0+ earthquakes from 2002 to 2018 using a time interval of 15 minutes. The curve is shown in normal style (a) and in colorful style (b), respectively. We use polar coordinates in (b) and show the stacking number around the Earth with a 24-hour-clock system. The daily stacking number of earthquakes ranges from 3331 to 3796. The dashed line denotes the reference number of 3500.

We further stacked the catalog using different periods to identify the uniqueness of the dominant period. Figure 5 shows the stacking results using 5 hours + 40 minutes, 6 hours and 6 hours + 20 minutes. Obviously, the stacking results using 6 hours exhibit the strongest coherency. Figure 6 further shows the stacking results using different periods from 5 hours to 7 hours with a time interval of 15 minutes. Again, the peak coherency appears at 6 hours. Therefore, we conclude that the dominant period of the time sector from 0:00 to 6:00 is of 6 hours. This means that the earthquake may have a dominant diurnal period for the whole catalog, at least from midnight to daybreak. To exhibit the daily behavior of the M1.0+ Japanese earthquakes, we show the daily stacking result using a 15-minute time interval in Fig. 7. Note that only the time sector from 0:00 to 6:00 is reliable, where the peak locates around 1:30 to 2:30, and a smaller peak appears around 4:30. Between them, the peak valley is also obvious.

### Reliable local peaks in stacking results

According to Atef *et al.* [22], the 24-hour period could be caused by humans’ daily activities, such as the subway, ground traffic and industrial productions. These cultural noises may apparently reduce the detectability of weak earthquakes, whereas it is difficult to explain the 12-hour period extracted here. In addition, the peak value of the stacking result within the reliable time sector of 0:00 to 6:00 appears at about 1:30, and the earthquake number shows an overall descending trend from 0:00 to 6:00. These results are consistent either using a time interval of 1 hour (Fig. 3 or Supplementary Fig. 2, available as Supplementary Data at *NSR* online) or 15 minutes (Fig. 7) when using various lower limits of earthquake magnitude (from M1.0 to M4.0 with an interval of M0.5). This also indicates that our method is not sensitive the magnitude completeness, since there is no apparent difference when using the lower limits of M1.0 [26], M2.5 and M3.0 [25]. Furthermore, if the detectability of the seismic network were affected by human activities, one would expect a peak around 3:00 to 4:00, rather than 1:30, since human activities usually are lowest at this time. On the contrary, the peak valley just arises around 3:00 to 4:00 in our stacking results. This conflict verifies that the peaks in the stacking results are reliable and they reflect the fundamental rule of earthquakes.

### Possible relationship between earthquakes and the Sun

Both the ocean tide and synthetic earth tide exhibit dominant periods at 12 and 24 hours as well as their neighbors (Fig. 2e), which are associated with the Sun and the Moon, respectively. In contrast, the earthquake catalog exhibits only dominant periods at 12 and 24 hours (Fig. 2e). Surprisingly, these two observed periods are both associated with the Sun rather than the Moon, compared with the Japanese ocean tide and earth tide (Fig. 2d and e). The fact that there is no correlation between earthquakes and the Moon may initially seem absurd, since the Moon mainly controls both ocean tides and earth tides. Calculations of the synthetic earth tide [30], intentionally ignoring the Moon, exhibit similar patterns to earthquakes. This confirms that the extracted dominant periods of Japanese earthquakes are only associated with the Sun (Fig. 2e). Therefore, we posit that the dominant period of earthquakes is mainly associated with the Sun, not the Moon. This may imply that earthquakes have a completely different mechanism compared with the ocean tides and earth tides, although they are all influenced by the celestial bodies.

## DISCUSSION

Based on the above observations and numerical experiments, we argue that there is a unique mechanism linking Japanese earthquakes and the Sun. This mechanism is likely not dominated by universal gravitation, since the observed periodicities are not consistent with the Moon's tidal periods. Aside from universal gravitation, one of the main differences between the Sun and the Moon is that the Sun is continuously releasing electromagnetic radiation and solar wind into space. It is well known that sunlight can induce large-scale daily and seasonal changes in the atmospheric temperature on the Earth. In addition, Zhan and Shearer [31] reported the possible seasonality in large deep-focus earthquakes. Some unclear factors from the Sun should be the main source of possible earthquake triggering. Of course, it is also possible that the Earth's rotation or the daily variation of the Earth's interior play an important role in triggering different number of earthquakes at different time sectors. Future work will require the integration of space physics, seismology and physics of the Earth's interior to solve the mysterious origin of earthquakes. In addition, we need to accumulate more earthquake data in future, both in Japan and in other regions, to verify the dominant period and to extract more weak periods from the catalog. We also need to develop some new techniques to extract the other periods from strong background noise, especially for possibly existing weak components associated with the Moon, which could provide more support in discovering more basic rules of earthquakes.

Small earthquakes are the majority of the earthquake catalog; thus, the extracted period can only reflect the rule of small earthquakes. Our method can not statistically determine the period of big earthquakes, which strongly affect human's activities, due to the too limited number of big earthquakes. The extracted period is only meaningful in terms of statistics, since it is basically fairly weak. In other words, our method can not predict any earthquake and can not infer the seismic activities in a particular area.

## CONCLUSION

In this paper, we identified two periodicities of 12 and 24 hours by analysing the Japanese earthquake catalog using time-frequency analyses and stacking. To the best of our knowledge, these two periodicities are the shortest in the literature, which are much smaller than the previous shortest periodicity of 2 weeks reported by others recently. Surprisingly, these two periodicities of earthquakes are only associated with the Sun rather than the Moon. The synthetic earth tides, after intentionally ignoring the contribution from the Moon, present similar periodicities to the earthquakes. The daily stacking number of earthquakes using a 15-minute or 1-hour interval shows a peak around 1:30 rather than the usually expected 3:00 to 4:00; interestingly, the peak valley just appears around 3:00 to 4:00. This trend is very consistent for various lower limits of earthquake magnitude from M1.0 to M4.0. Furthermore, bigger earthquakes show more evident variations in the stacking results within the reliable time sector of 0:00 to 6:00. Our work suggests that the earthquakes have a dominant diurnal period, at least from midnight to daybreak, which could be helpful to opening a new window to explore the physical mechanism of earthquakes.

## METHODS

### Correcting the original time of earthquakes to Local Mean Time

As shown in Fig. 1b, the M1.0+ Japanese earthquakes under the mainland ranges from 128° to 147° in longitude, where the maximum deviation from 135° (the center of Japan Standard Time) is up to 12°, which is close to the longitude difference between two adjacent time zones (i.e. 15°). Therefore, we should correct the original time to Local Mean Time, which can faithfully reflect the azimuth of the Sun in the sky. The time correction can be calculated by $\Delta t = ( {l - {l_0}} ) \times 3600/15,$ where $l$ is the longitude of the earthquake, ${l_0}$ is the longitude at the center of the time zone (for Japan Standard Time, ${l_0} = {135^ \circ })$ and $\Delta t$ is the time correction (in seconds). The calculated time correction $\Delta t$ should be added to the original time.

### Time-frequency analyses

Time-frequency analyses are powerful tools for extracting the instantaneous signal frequency, especially when there are strong background noises. Time-frequency analyses first fetch some samples (e.g. 1024) from the whole data set using a time window; then, they perform local spectrum analyses within this window; next, they moves the window slightly forward (e.g. 512 samples or less); and, finally, they repeat this process until the whole data series has been analysed. Time-frequency analyses can help us to identify the dominant periods within each window; thus, we can observe a significant trend in the spectrum if the spectrum in each window is similar to its neighbors. This method requires that the input data are evenly sampled over time; otherwise, strong artifacts would appear in the results. If the data are unevenly sampled or some samples are missing, we need an interpolation to reduce artifacts.

The number of earthquakes in each time interval behaves as a random signal; thus, it is fairly difficult to interpolate the data. If only a few records are null, we can simply set them to zero instead; however, if a large number of records are null, we would have strong artifacts in the results. The catalog of earthquakes in Japan only has a few null records after we count the number of earthquakes within each hour. Figure 2b shows the results of time-frequency analyses on M0.5+ earthquakes under the Japanese mainland with a time interval of 1 hour, which shows a clear dominant period at 24 hours.

### Spectrum stacking

Time-frequency analyses can only present a weak trend in the spectrum. We can perform spectrum stacking along time windows and we can obtain a stacked spectrum, which could have a much higher signal-to-noise ratio; thus, it is helpful to improve the resolution and reliability of the extracted period, as shown in Fig. 2e.

### Periodic stacking

If we know the dominant period, we can perform a catalog stacking accordingly, as shown in Fig. 3 and Supplementary Fig. 2, available as Supplementary Data at *NSR* online. We can manually tune the stacking window around the dominant period, which can verify the uniqueness of the dominant period, as shown in Fig. 5. For a set of completely random data (without any period), we would have a nearly flattened trend when we use various stacking windows. However, if there is any period existing in the data, even though its amplitude is fairly weak compared with the strong background noise, the stacking method can extract the dominant period only if the accumulated catalog is long enough.

## Supplementary Material

nwy117_Supplemental_File
