# Supplementary material for: Evidence for diurnal periodicity of earthquakes from midnight to daybreak
Source: Natl Sci Rev. 2018 Oct 8;6(5):1016–23. doi: 10.1093/nsr/nwy117 (PMC8291617; doi:10.1093/nsr/nwy117)
Supplement: nwy117_Supplemental_File [file nwy117_supplemental_file.docx]

Evidence for diurnal periodicity of earthquakes from midnight to daybreak

Supplementary Information

Verifying the 24-hour period with synthetic data

We perform a numerical experiment using a noisy synthetic catalog of earthquakes to verify the 24-hour period extracted from the real data set. First, we generate a cosine function with 24-hour period (Fig. S1c); then, we generate two random series: one is dense (Figs. S1b) and the other is sparse (the spike in Fig. S1b or spikes in Fig. S1d); finally, these three series are combined with different amplitudes, which are generally consistent with the real data in Japan. We set the amplitude for the daily variation being 1. In contrast, the maximum amplitude of noisy background is 20 and the maximum amplitude of sparse spikes is up to 200. The weak 24-hour period can be clearly extracted even though its amplitude (i.e., 1) is far smaller than the background noise (~20). This experiment shows that the time-frequency analyses are powerful for extracting weak periods from the dataset with strong background noises. Therefore, the extracted 24-hour period in the real earthquake data in Japan is reliable for subsequent analyses and discussion.

In fact, the time-frequency analyses can extract a much weaker signal (i.e., with a smaller amplitude of 0.5), since the stacked spectrum shown in Figure S1f is still evident enough. Compared with the stacked spectrum of real earthquake data shown in Figure 2e, we can deduce that the actual noise level should be much higher than we expected.

**
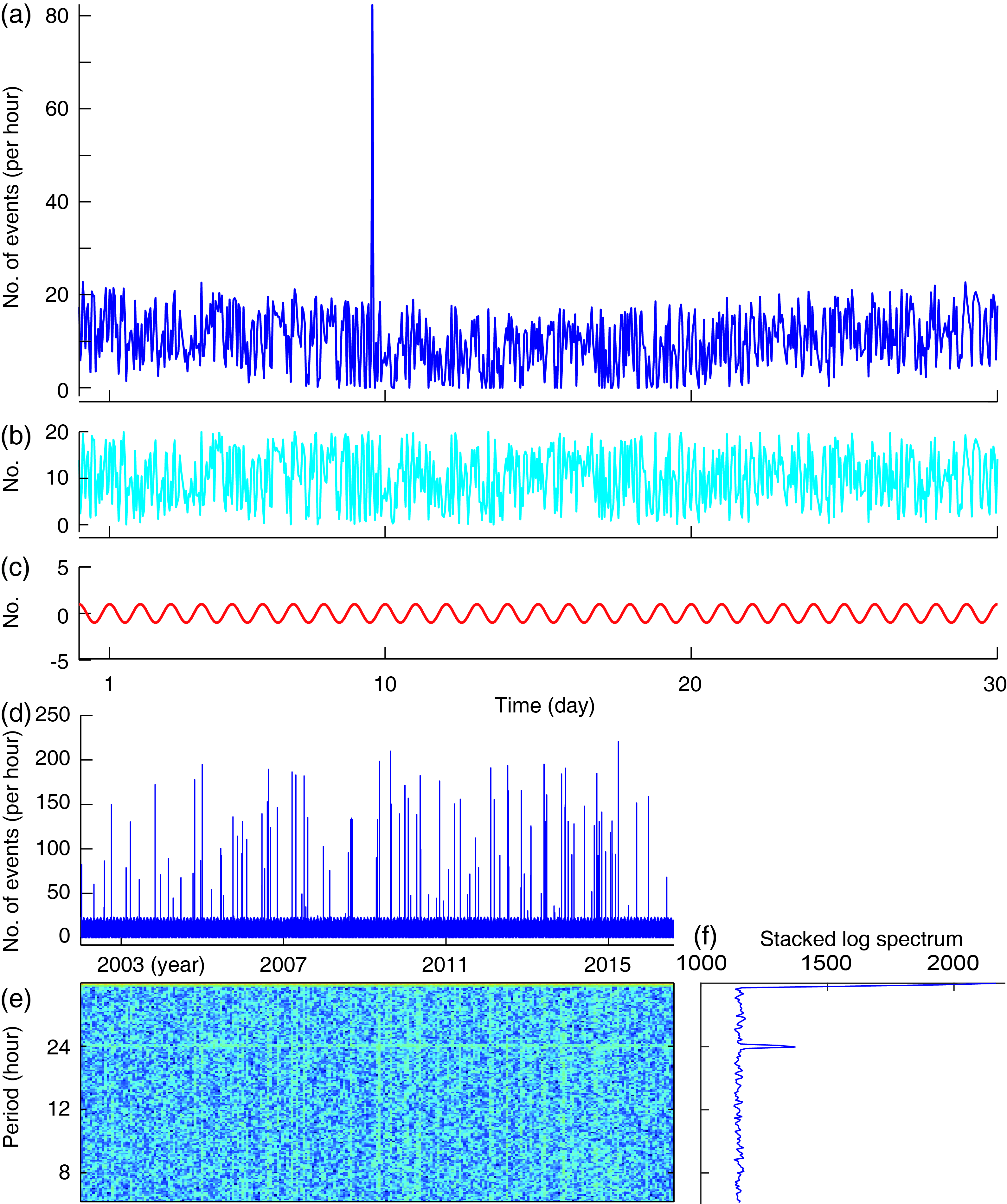
**

**Figure S1.** Time-frequency analyses on the synthetic catalog of earthquakes. A random number of earthquakes (the blue curve in (a)) has three components: a dense random series with a maximum amplitude of 20 (the aqua curve in (b)), a 24-hour period with a normalized amplitude of 1 (the red curve in (c)), and a sparse random series with random amplitude from 10 to 200 (the spike in (a) or spikes in (d)). (d) The whole synthetic data; (e) the time-frequency spectrum; (f) the stacked spectrum.

**
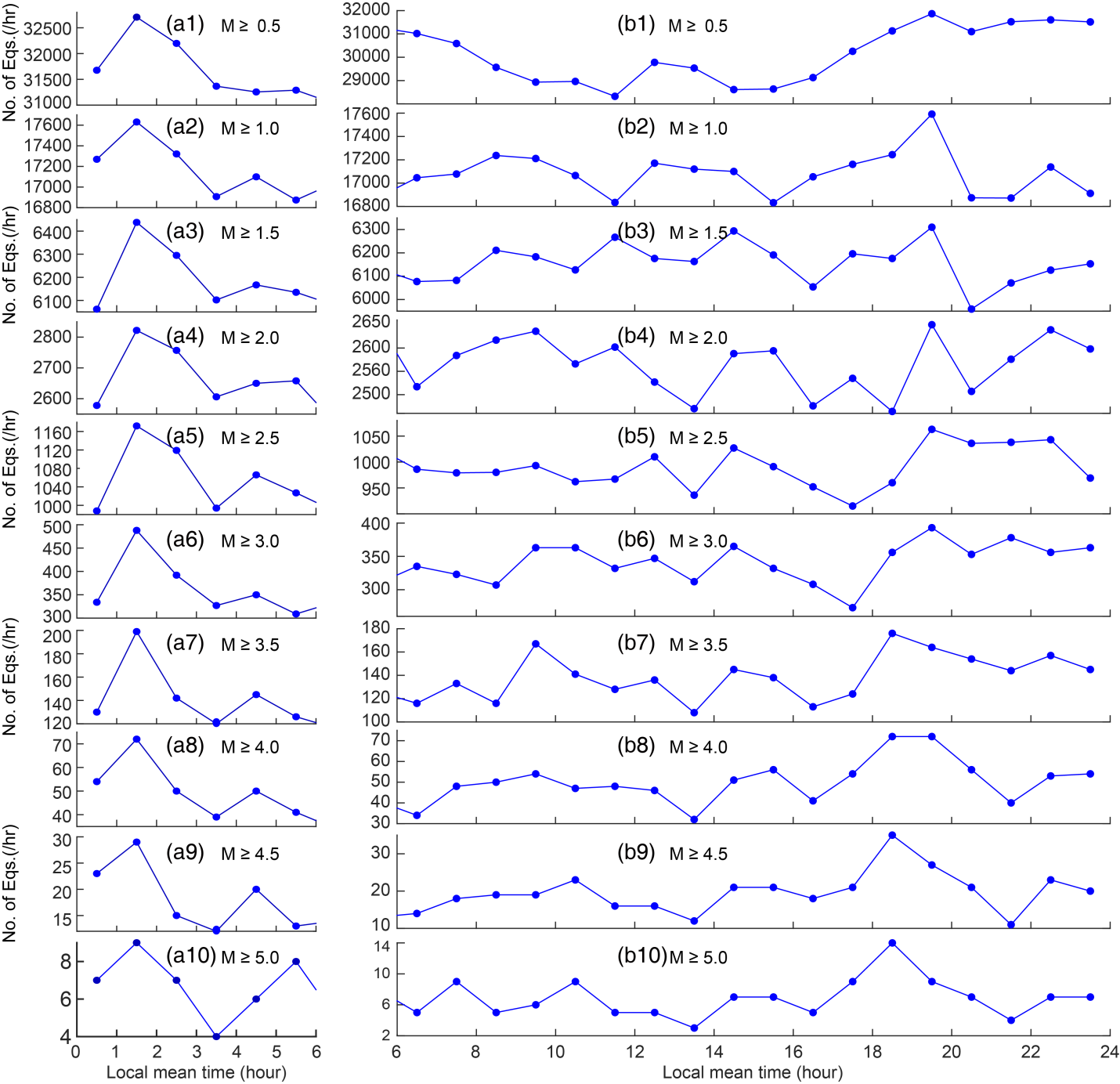
**

**Figure S2.** Daily stacking number of Japan earthquakes from June 2002 to July 2018 using a time interval of 1 hour. (a) time sector of 0:00 to 6:00; (b) time sector of 6:00 to 24:00. The lower limit magnitude of earthquake for daily stacking is shown in each subfigure.
